# Modeling and Prediction of Oyster Norovirus Outbreaks along Gulf of Mexico Coast

**DOI:** 10.1289/ehp.1509764

**Published:** 2015-11-03

**Authors:** Jiao Wang, Zhiqiang Deng

**Affiliations:** Department of Civil & Environmental Engineering, Louisiana State University, Baton Rouge, Louisiana, USA

## Abstract

**Background:**

Oyster norovirus outbreaks often pose high risks to human health. However, little is known about environmental factors controlling the outbreaks, and little can be done to prevent the outbreaks because they are generally considered to be unpredictable.

**Objective:**

We sought to develop a mathematical model for predicting risks of oyster norovirus outbreaks using environmental predictors.

**Methods:**

We developed a novel probability-based Artificial Neural Network model, called NORF model, using 21 years of environmental and norovirus outbreak data collected from Louisiana oyster harvesting areas along the Gulf of Mexico coast, USA. The NORF model involves six input variables that were selected through stepwise regression analysis and sensitivity analysis.

**Results:**

We found that the model-based probability of norovirus outbreaks was most sensitive to gage height (the depth of water in an oyster bed) and water temperature, followed by wind, rainfall, and salinity, respectively. The NORF model predicted all historical oyster norovirus outbreaks from 1994 through 2014. Specifically, norovirus outbreaks occurred when the NORF model probability estimate was > 0.6, whereas no outbreaks occurred when the estimated probability was < 0.5. Outbreaks may also occur when the estimated probability is 0.5–0.6.

**Conclusions:**

Our findings require further confirmation, but they suggest that oyster norovirus outbreaks may be predictable using the NORF model. The ability to predict oyster norovirus outbreaks at their onset may make it possible to prevent or at least reduce the risk of norovirus outbreaks by closing potentially affected oyster beds.

**Citation:**

Wang J, Deng Z. 2016. Modeling and prediction of oyster norovirus outbreaks along Gulf of Mexico coast. Environ Health Perspect 124:627–633; http://dx.doi.org/10.1289/ehp.1509764

## Introduction

Norovirus is a highly infectious and costly pathogen (Atmar 2010; Wang and Deng 2012). It is the leading cause of endemic diarrheal disease across all age groups, and the cause of half of all gastroenteritis outbreaks worldwide (Hall 2012). At this time, little can be done to prevent human health risks of norovirus because there is no vaccine to prevent infection, no drug to treat it, and no model to predict norovirus outbreaks. However, it might be possible to prevent or at least reduce norovirus infection and associated costs by monitoring norovirus indicators daily and predicting norovirus outbreaks in advance, if environmental factors that predict the outbreaks can be identified and modeled.

Norovirus is commonly found in oysters growing in contaminated waters. Oyster contamination with norovirus may occur after heavy rainfall, which often results in contaminated overland runoff, combined sewer overflow, or hydraulic overload in sewage treatment plants (Le Guyader et al. 2006; Miossec et al. 2000; Yang et al. 2012; Ye et al. 2014). Oysters filter large volumes of water as part of their filter-feeding activities and are able to accumulate and concentrate norovirus (Le Guyader et al. 2010). Therefore, oyster contamination with norovirus may cause norovirus outbreaks. Although environmentally mediated norovirus outbreaks do not occur frequently, outbreaks may have substantial impacts on human health, with symptoms including diarrhea, nausea, vomiting, and abdominal cramps (Le Guyader et al. 2010). In addition, secondary transmission from person to person may occur, and outbreaks may result in school and workplace closures, as well as the closure of oyster harvesting waters and costly oyster recalls. Oyster norovirus outbreaks have been reported worldwide. Westrell et al. (2010) reported 334 cases in 65 clusters of oyster norovirus outbreaks from January through March 2010 in five European countries, including the United Kingdom, Norway, France, Sweden, and Denmark. A total of 305 cases were attributed to oyster norovirus outbreaks that occurred from 16 December 2003 to 4 January 2004 in Singapore (Ng et al. 2005). Multiple clusters of norovirus outbreaks were associated with raw oyster consumption affecting 36 people in British Columbia, Canada, in 2010 (McIntyre et al. 2012), and 525 cases were identified in March 2013 in Australia from consumption of norovirus-contaminated oysters (Lodo et al. 2014).

Oysters are filter feeders that pump a large amount (about 5 L/hr) of water (including norovirus in the water) through their gills and mantle (Wang and Deng 2012). Oyster norovirus outbreaks appear to be influenced by environmental factors, such as rainfall, temperature, and salinity (Wang and Deng 2012). Norovirus outbreaks in oyster harvest waters often take place during cold weather (Maalouf et al. 2010). Wastewater effluent from failing wastewater treatment plants may be a common route of norovirus transmission after heavy rainfall (Flannery et al. 2012, 2013; Schijven et al. 2013). Maalouf et al. (2010) found that viruses tend to attach to fine sediment particles or silts, and that salinity may enhance the binding of viruses to fine sediment particles. As a result, viruses are protected by marine sediment and may persist in an infectious state for several months, especially when salinity is low. In oyster harvest waters, norovirus survival rates increase with reduced exposure to ultraviolet light (Lee and Ko 2013). A 1-month follow up study suggested that strong winds were associated with a rapid increase in norovirus prevalence in oyster harvest areas (Grodzki et al. 2012). However, despite recent progress in identifying individual environmental risk factors for norovirus outbreaks, little is known about key environmental factors controlling the outbreaks.

The overall goal of the present study was to establish a quantitative model for predicting oyster norovirus outbreaks. Our specific objectives were *a*) to identify environmental predictors for oyster norovirus outbreaks, and *b*) to construct a model for estimating the probability of an oyster norovirus outbreak.

## Materials and Methods

Due to the lack of concentration data for norovirus in oyster-growing waters, we developed a probability-based Artificial Neural Network (ANN) model, using historical data on norovirus outbreaks in 30 oyster harvest areas from 1994 through 2007, for predicting the risk of potential oyster norovirus outbreaks in a probabilistic fashion, similar to weather forecasts.

### Study Area

**Figure 1:**
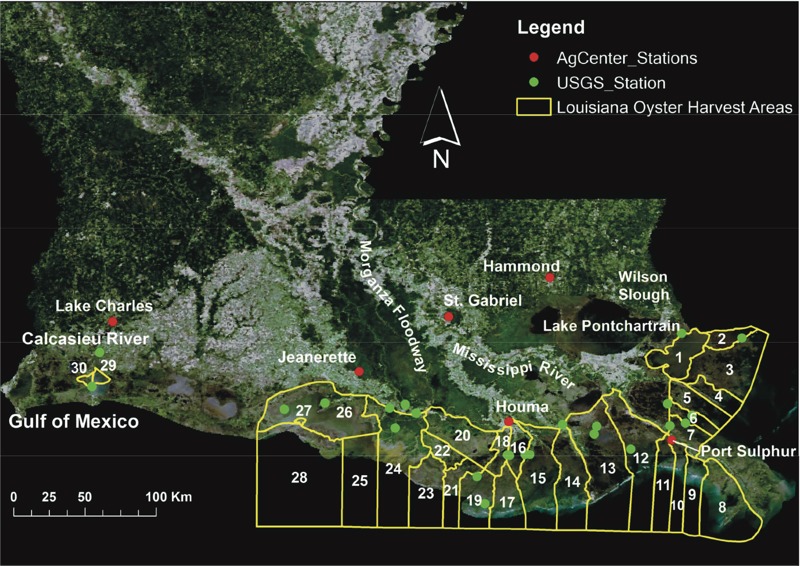
Oyster harvesting areas along Louisiana coast, USA. [MODIS Surface Reflectance image from 2001 was retrieved from https://ladsweb.nascom.nasa.gov/ maintained by the NASA EOSDIS Level 1 and Atmosphere Archive and Distribution System Distributed Active Archive Center (LAADS DAAC), NASA’s Goddard Space Flight Center, Greenbelt, Maryland. The data product for the image was provided by NASA.]

Louisiana is one of the primary oyster-producing states in the United States. There are 30 oyster harvest areas along the Louisiana coast (Figure 1). Areas 1–7 are located to the south of Lake Pontchartrain and to the east of the Mississippi River, whereas Areas 8–30 are located west of the river. Areas 29 and 30 are actually in the Calcasieu River estuary. In addition to fresh water discharges from the rivers and lakes, the oyster-harvesting areas may also receive sewage from oyster-harvesting boats and failing septic systems along the shoreline and contaminated urban stormwater runoff and combined sewer overflows from nearby cities, constituting potential sources of oyster norovirus contamination (Schaeffer et al. 2013).

### Outbreak Data Sets

Historical norovirus outbreaks in Louisiana oyster harvest areas were recorded in Louisiana morbidity reports released annually by Louisiana Department of Health and Hospitals (Table 1). Some additional data for individual outbreaks, such as the duration and the number of infected people, were provided by Ronald J. Dugas, at the Louisiana Department of Wildlife and Fisheries (personal communication).

**Table 1:** Norovirus outbreaks during 1994–2013.

Norovirus outbreak period	Areas
25 January 1996–16 February 1996	6, 7
8 February 1996–23 February 1996	6, 7
22 December 1996–3 January 1997	6, 7
1 March 2002–31 March 2002	1
12 March 2002–28 March 2002	6, 7
10 December 2007–21 December 2007	3
20 March 2010–25 March 2010	3
6 March 2010–24 March 2010	7
27 March 2010–30 March 2010	13
26 April 2012–8 May 2012	23
28 December 2012–4 January 2013	30
Norovirus outbreak data were collected from Louisiana Morbidity Reports (http://dhh.louisiana.gov/index.cfm/newsroom/archives/126).	

*Data and sources for environmental variables.* Environmental data for five environmental predictors were collected for the years 1994–2014. Because the number of norovirus outbreak events is limited, the 21 years of data were split into two data periods based on time sequence of norovirus outbreaks: period 1 (1994–2007) used for model development, and period 2 (2008–2014) for prediction (independent testing or cross-validation). Data for gage height, water temperature, and salinity were obtained from U.S. Geological Survey (USGS) stations (http://www.usgs.gov/) located in Louisiana oyster harvest areas, and data for rainfall and wind (including wind speed and direction) were obtained from Louisiana State University Agricultural Center (LSU AgCenter) stations (http://weather.lsuagcenter.com) (Figure 1).

Gage height is essentially the water depth in an oyster-growing area. We selected gage height as a potential predictor because low gage height (low water depth) may reduce dilution of sewage-contaminated runoff from antecedent rainfall, thus potentially increasing norovirus concentrations in oyster-growing waters (Wang and Deng 2012). We selected water temperature as an environmental predictor because cold weather or low temperature favors norovirus survival (Westrell et al. 2010). Salinity has also been reported to affect the persistence of norovirus in oyster harvest waters by enhancing virus binding to fine sediment particles (Maalouf et al. 2010). We also selected rainfall as a potential predictor because it facilitates the transmission of norovirus from inland sources to oyster-growing waters (Wang and Deng 2012). Finally, we included wind as an environmental predictor, defined as a function (product) of both wind speed and wind direction. Generally, an onshore wind causes water levels to rise at the coast (set-up), whereas an offshore wind causes water levels to fall (set-down). We hypothesized that offshore winds would facilitate the transmission of norovirus from land to oyster harvest waters, in addition to causing water levels to fall, thus increasing the concentration of norovirus in oyster-growing waters. Because the wind direction that determines whether the wind is onshore or offshore varies among different locations, we defined a dichotomous indicator variable (1 if offshore, 0 if onshore) separately for each location. Specifically, winds blowing from 180 to 360 degrees were defined as offshore in oyster harvest Areas 1–7, winds from 90 to 270 were classified as offshore in Areas 8–17, and winds blowing from 0 to 180 degrees were classified as offshore for Areas 18–30. It should be noted that wind, as the product of speed and direction, is not a dichotomous variable.

Due to the sparse distribution of LSU AgCenter weather stations, several oyster-harvesting areas share the data from a common nearby weather station. Specifically, the rainfall and wind data from Hammond station are used for Areas 1–3, and data for Areas 4–7, 8–19, 20–26, and 27–30 are collected from the stations in the cities: Port Sulphur, Houma, Jeanerette and St. Gabriel, and Lake Charles, respectively.

Because a USGS station is located at the border between Areas 2 and 3, the two areas (2 and 3) share the same data from the USGS station. Due to missing data in some years for Area 24, the same data sets are used for Areas 24 and 26. Likewise, Areas 29 and 30 are also treated as a single area in terms of data sharing. As a result, there are only 12 oyster harvest areas where independent data are available, including Areas 1, 2 (including 3), 6, 7, 12, 13, 14, 15, 17, 19, 24 (including 26), and 30 (including 29). In areas containing two or more USGS stations, the station with the most complete data was used as the primary station, and the other stations were used as a source of supplementary data. All data are available online at the hourly time scale, making it possible to obtain the daily maximum, daily minimum, daily change, and daily average of the variables, which are needed in the predictive model.

*Selection of model input variables.* Stepwise regression analysis has been widely used to select model input variables (predictors) and reduce the number of potential model input variables for ANN analysis (Genell et al. 2010; Zhang et al. 2012, 2015). We used forward stepwise regression (PROC REG, SELECTION = STEPWISE; SAS 9.2) (SAS Institute Inc. 2008) to select a final set of model predictors of norovirus outbreaks based on both the partial *R* ^2^ values for individual variables, and the overall model *R* ^2^ values. We began with a large pool of potential environmental predictors that included several measures of gage height [daily maximum gage height (daily maximum gage height – daily minimum gage height), minimum gage height, average gage height, and daily change in gage height], water temperature (daily average temperature, minimum temperature, and maximum temperature), rainfall (rainfall on the same day, and rainfall on 1–15 previous days), salinity (daily average, maximum, and minimum), and wind (direction and speed). Environmental variables selected for the final model, ranked from the highest to lowest individual *R* ^2^ values, were maximum gage height, minimum temperature, wind (offshore or onshore, as defined above), change in gage height, rainfall 9 days before, and minimum salinity, with all variables except rainfall defined by values on the current day. Variables were automatically selected in the stepwise regression analysis based on individual *R* ^2^ *p*-values < 0.05 except for salinity (*p* = 0.15), which was included because it improved the overall model *R* ^2^ value by 9.84%.

*Normalization of ANN model input variables (environmental predictors).* After selection, all model input variables were normalized to a range of 0–1 using Equation 1, where *a* represents a model input variable, max(*a*) and min(*a*), which refer to observed historical maximum and minimum values of *a*, respectively. The parameter, *N*(*a*), stands for the normalized variable of *a*.


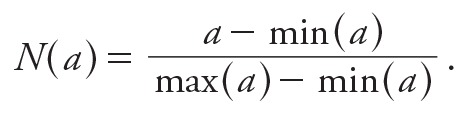
.[1]

**Figure 2:**
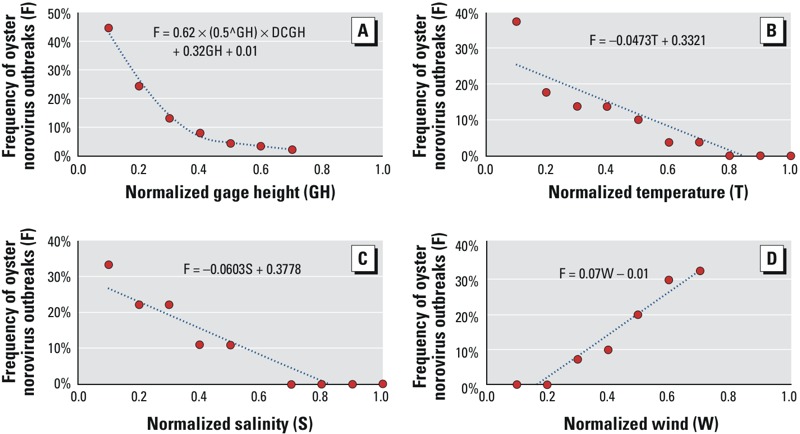
Relationships between the frequency distribution of norovirus outbreaks (*y-*axis) and normalized environmental predictors (*x-*axis): (*A*) gage height (GH) and daily change in gage height (DCGH), (*B*) temperature (T), (*C*) salinity (S), and (*D*) wind (W).

After normalization, we graphically examined the relation between each environmental predictor (except rainfall, which had a limited distribution) and the frequency of norovirus outbreaks (Figure 2). Figure 2A shows that the frequency of norovirus outbreaks has a highly nonlinear relationship with the normalized gage height (GH) and daily change in gage height (DCGH). Basically, about 70% of historical norovirus outbreaks occurred when GH was lower than 0.2. For this reason, the variable (0.5^GH^)DCGH (selected through a trial-and-error procedure to produce the best fit curve in Figure 2A) was actually used as a model input variable in lieu of the daily change in gage height while GH was also included as another model input variable. The nonlinear relationship was derived by using the Microsoft Excel LINEST function for multivariate regression with the frequency of norovirus outbreaks as the dependent variable and the predictors (0.5^GH^)DCGH and GH as independent variables. Temperature, salinity, and wind had approximately linear relations with the frequency of norovirus outbreaks (Figure 2B–D) and were therefore modeled as normalized continuous variables. The linear relationships were also derived using the Excel LINEST function. However, the values of variables that were negatively associated with outbreaks were subtracted from 1 so that all predictors would be positively associated with the probability of an outbreak, such that the final set of normalized predictors used as input for the ANN model were 1 – gage height, (0.5^gage height^) × daily change in gage height, 1 – temperature, 1 – salinity, wind, and rainfall 9 days prior.

The ANN was previously found to be a simple yet effective tool for describing nonlinear relationships between the concentration of fecal indicator bacteria in coastal recreational waters and environmental variables (Zhang et al. 2012, 2015). A significant advantage of an ANN model over numerical models is its flexibility in dealing with a large number of model input variables when the specific functional relationship between a dependent variable and independent model input variables is unknown (Hamilton et al. 1997), as is the case for norovirus outbreaks. We used the ANN Toolbox in the MATLAB Program (version 2010a) to train a feed-forward ANN model using the error back-propagation algorithm to integrate the environmental predictors for the prediction of oyster norovirus outbreaks. The ANN model architecture consists of an input layer with the six predictor variables, a hidden layer with 20 neurons, and an output layer that displays the estimated probability of oyster norovirus outbreaks based on the model. In the model development phase, the normalized data sets for the six input variables, collected from 1994 through 2007, were employed in the input layer. The 14 years of data from the 12 oyster-harvesting areas with complete and independent data sets (Areas 1, 3, 6, 7, 12, 13, 14, 15, 17, 19, 24, and 30) were first combined without distinguishing one area from another, and were then randomly split into three groups for training (60% of data), validation (20% of data), and testing (20% of data). The data values in output layer were either 1 (if there was an oyster norovirus outbreak) or 0 (if there was no outbreak). We identified the best-trained ANN model based on the performance of top-ranked models in reproducing confirmed oyster norovirus outbreaks. Predictions based on the ANN model were compared with historical data for norovirus outbreaks to identify threshold values for model-predicted probabilities that were consistently associated with outbreaks. We subsequently refer to the best-trained ANN model as the Norovirus Outbreak Risk Forecasting model, or NORF model.

*Sensitivity analysis.* To evaluate the sensitivity of the NORF model to individual model parameters, we changed the mean value of each input variable (gage height, temperature, salinity, wind, and rainfall), one at a time, by ±5%, ±10%, ±20%, ±30%, and ±40%, respectively, and calculated the percent change in the model output (i.e., the predicted probability of an outbreak) with each incremental change in the individual predictor variables.

*Independent cross-validation.* A cross-validation was performed to measure the predictive ability of the model using data collected from 2008 to 2014 that were not used in the model development phase. The primary criterion for assessing model performance during the cross-validation phase was to accurately predict all past oyster norovirus outbreaks from 2008 to 2014 without producing false outbreaks.

*Predictions for another location.* In addition to Louisiana, the NORF model was also tested as part of the cross-validation using the data collected from the Copano Bay, Texas, oyster-harvesting area for 1 January–31 December 2013. The data for gage height, temperature, and salinity were collected from USGS station 8211503, and rainfall data were collected from USGS station 8288570. Wind speed and direction data were collected from NOAA station 8774513. Winds blowing from 67.5 to 247.5 degrees were defined as offshore winds. The data were processed using the same methods as those for processing Louisiana data. NORF model predictions of potential oyster norovirus outbreaks in this area were made for 365 days in 2013.

## Results

**Figure 3:**
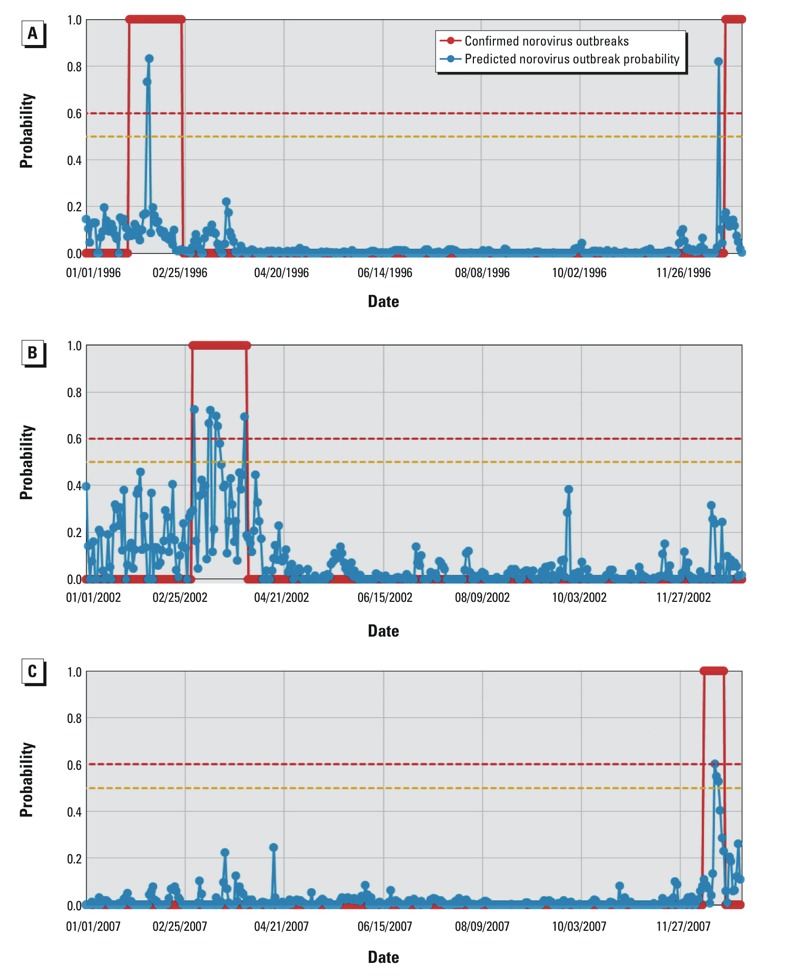
Comparison between the NORF model–predicted probabilities of norovirus outbreak and the observed norovirus outbreak probabilities (0 or 1) in oyster-harvesting areas along Louisiana Gulf Coast: (*A*) Areas 6 and 7 with outbreaks in February and December 1996, (*B*) Areas 1, 6, and 7 with outbreaks in March 2002, and (*C*) Area 3 with an outbreak in December 2007 The red horizontal line denotes the threshold probability of 0.6 for norovirus outbreaks, implying that a norovirus outbreak would occur if the model-predicted probability is > 0.6. Likewise, the yellow horizontal line indicates the threshold probability of 0.5 for non-outbreak, meaning that there would be no norovirus outbreaks if the model predicted probability is < 0.5.

We compared predicted probabilities of norovirus outbreaks based on the NORF model with the occurrence of reported outbreaks in 1996, 2002, and 2007 and identified a threshold model–based probability of 0.6 that consistently predicted the observed outbreaks (Figure 3).

Two oyster-harvest areas (6 and 7) were shut down on 16 and 23 February 1996, respectively, and oysters harvested in Area 6 from 25 January and Area 7 from 8 February were recalled. The NORF model predicted high norovirus outbreak probabilities of 0.733 for 4 February and 0.833 for 5 February, respectively (Figure 3A). There was another reported norovirus outbreak in Areas 6 and 7 in December 1996 that caused multiple clusters of illnesses (total *n* = 493) associated with the consumption of norovirus-contaminated raw oysters on 25 December (http://www.outbreakdatabase.com/details/louisiana-oysters-1996/). The two areas were closed on 3 January 1997, and oysters harvested between 22 December 1996 and 3 January 1997 were recalled. The NORF model predicted an outbreak probability of 0.820 for 18 December 1996. The model prediction suggests that oysters should have been recalled on 18 December instead of 22 December 1996 to 3 January 1997. The NORF model also predicted norovirus outbreaks that occurred in Areas 1, 6, and 7 in March 2002 (Figure 3B). In 2007, oysters harvested in Area 3 between 10 and 21 December were recalled due to a norovirus outbreak, while the NORF model predicted a norovirus outbreak with a probability of 0.604 for 16 December 2007 (Figure 3C).

In general, NORF model predictions were consistent with the reported oyster norovirus outbreaks, though there were some differences in the timing of the predicted versus reported outbreak dates. However, the reported onset dates for norovirus outbreaks were usually estimated based on post-outbreak epidemiologic investigations, and it is possible that the reported norovirus outbreak onset dates or oyster recall dates may not be accurate. The NORF model was also run for other years during 1994–2007 that did not have any reported norovirus outbreaks. The model predicted low outbreak probabilities (< 0.5) during all of these years (results not shown).

### Sensitivity Analysis

**Figure 4:**
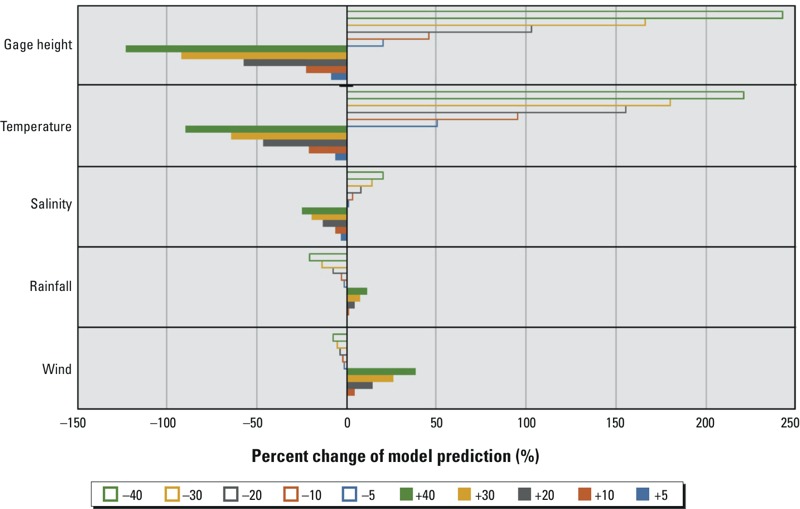
Sensitivity of the NORF model output to environmental predictors. Solid bars indicate percent changes in the probability (model output) predicted by the NORF model due to positive changes to model input variables; the hollow bars indicate percent changes in the model output due to negative changes to model input variables.

Decreases in gage height, temperature, and salinity, and increases in rainfall and wind, were associated with increases in model-predicted probabilities of oyster norovirus outbreaks (Figure 4). Likewise, increases in gage height, temperature, and salinity, along with decreases in rainfall and wind, were associated with lower estimated probabilities. Changes in gage height and temperature had the greatest influence on model predictions, whereas salinity, rainfall, and wind also affected model predictions. Specifically, the NORF model predicted norovirus outbreak probability increases of 20%, 45%, 103%, 167%, and 243% when the gage height was reduced by 5%, 10%, 20%, 30%, and 40%, respectively. Likewise, the model predicted norovirus outbreak probability increases of 51%, 95%, 155%, 180%, and 221% for corresponding decreases in temperature.

### Independent Cross-Validation

**Figure 5:**
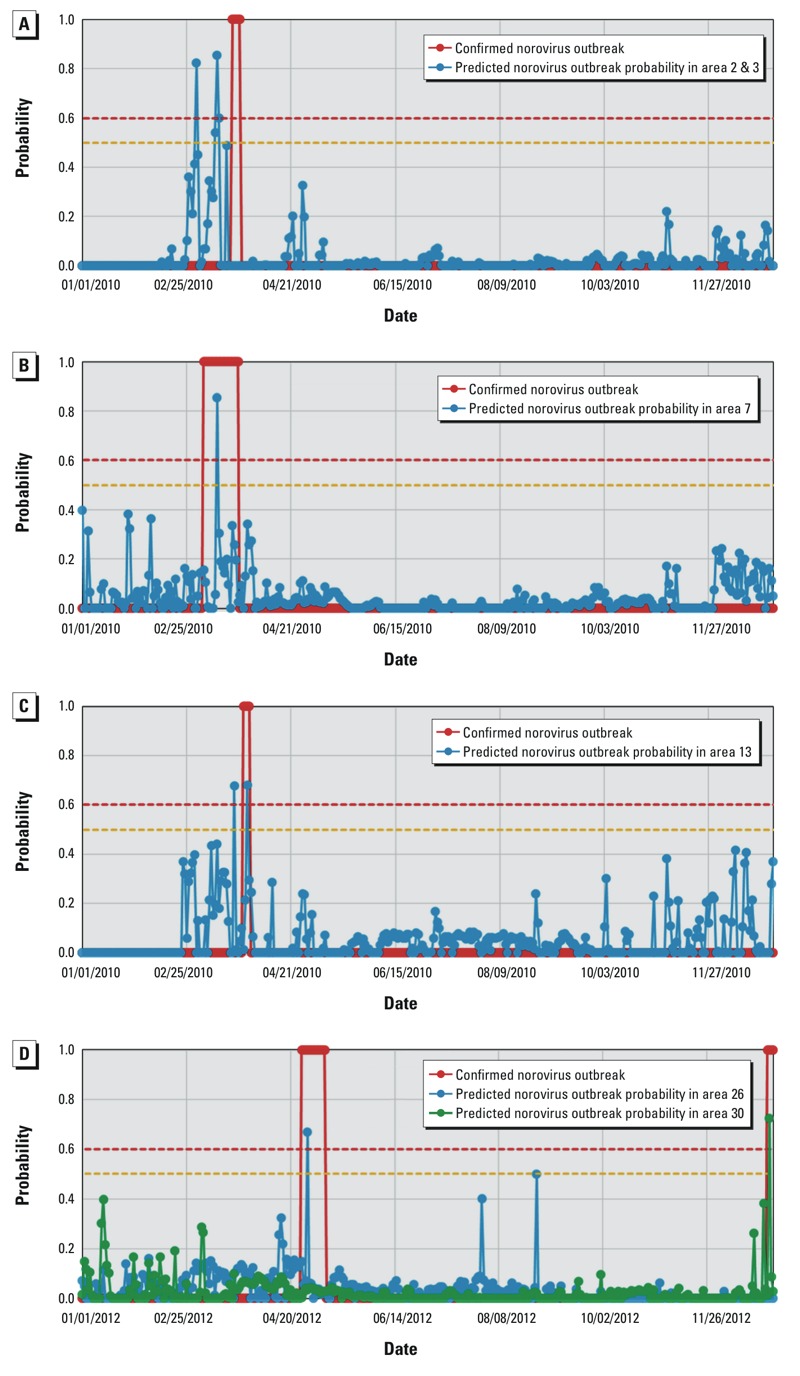
Comparison between the NORF model–predicted probabilities and the observed probabilities (0 or 1) of the norovirus outbreaks in oyster-harvesting areas along the Louisiana Gulf Coast: (*A*) Areas 2 and 3 with outbreaks in March 2010, (*B*) Area 7 with an outbreak in March 2010, (*C*) Area 13 with outbreaks in March 2010, and (*D*) Area 26 with an outbreak in April 2012 and Area 30 with an outbreak in December 2012.

There were five reported norovirus outbreaks in the independent testing period from January 2008 through December 2014 (Table 1). Three of the five outbreaks took place in March 2010. Area 3 was closed on 25 March after 14 people became ill due to the consumption of norovirus-contaminated raw oysters harvested between the suspected (unconfirmed) period of 20 March and 25 March (Figure 5A). Nine additional cases were reported after the closure of Area 3. The NORF model predicted at least two norovirus outbreaks on 2 March (probability = 0.82) and 13 March (probability = 0.85) to 14 March (probability = 0.60), respectively. The predicted outbreak on 2 March coincided with an extremely strong offshore wind (normalized value = 0.52, or 16.1 m/sec), low gage height (normalized = 0.09, 0.68 feet) and low temperature (normalized = 0.22, 11.2°C).

The model-predicted and reported outbreak dates (2 March vs. 20 March) differed for Area 3, but the model-predicted outbreak on 2 March for Area 3 was close to the date of a reported norovirus outbreak on 6 March in Area 7 (Figure 5B). This area was closed on 24 March after 14 people were infected by norovirus after eating raw oysters harvested between 6 March (date inferred from post-outbreak investigation) and 24 March. The NORF model predicted a norovirus outbreak on 13 March in Area 7 with a probability of 0.85. Environmental conditions that favored an outbreak in Area 7 on this date included an extremely low gage height (normalized value = 0.08, –0.23 feet), low salinity (0.12, 3.2 parts per thousand), and strong offshore wind (0.52, 16.1 m/sec). Area 13 was also closed on 30 March after 19 people became ill after consuming norovirus-contaminated raw oysters harvested between 27 March and 30 March 2010 (Figure 5C). The NORF model predicted two norovirus outbreaks in Area 13 on 22 and 29 March, respectively, both with a probability of 0.68.

Environmental conditions in Areas 12 and 13 are very similar, and the monitoring stations in the two areas are also very close to each other. Although there were no reported norovirus outbreaks in Area 12, the NORF model predicted outbreaks on 13 March (probability = 0.74), 22 March (probability = 0.64), and 29 March (probability = 0.71).

There were two reported norovirus outbreaks in April–May and December 2012 in Areas 23 and 30, respectively (Figure 5D). Area 23 was closed on 8 May 2012 after 14 people became ill with norovirus after eating oysters at a restaurant on 28 or 29 April. The Louisiana Department of Health and Hospitals ordered a recall of all oysters harvested from that area since 26 April 2012. Because there is no monitoring station in Area 23, the NORF model cannot be used to predict outbreaks in this area. The NORF model did predict a norovirus outbreak in Area 24/26 on 29 April 2012, (probability = 0.67). However, Areas 22, 24, and 26 were not open for oyster harvesting (Louisiana DHH Office of Public Health 2012) during this time period, so it was not possible for norovirus outbreaks to spread in these areas. Another reported norovirus outbreak occurred in Area 30 at the end of 2012 (Louisiana DHH Office of Public Health 2013). Area 30 was closed on 4 January 2013 after 12 people became ill with norovirus after eating oysters harvested from this area between 28 December 2012 and 4 January 2013. The NORF model predicted a norovirus outbreak in Area 30 on 29 December 2012 (probability = 0.72) (Figure 5D), consistent with the observed norovirus outbreak.

### Predictions for Another Location

The U.S. Food and Drug Administration (FDA) warned consumers not to eat oysters harvested between 26 December 2013 and 9 January 2014 from Copano Bay, Texas, after they were linked to a norovirus outbreak (FDA 2014) that caused six norovirus illnesses in Louisiana residents. The NORF model was employed to predict the probability of norovirus outbreak in Copano Bay from 1 January to 31 December 2013. The model predicted the highest probability of a norovirus outbreak on 29 December 2013 with a probability of 0.53, below the 0.6 threshold probability defined for Louisiana, but above the lower threshold of 0.50. This suggests that the NORF model may be applicable to oyster-growing areas beyond the Louisiana coast. However, new or site-specific definitions for wind direction (specifically offshore or onshore wind for each individual area) must be derived.

## Discussion

Although a norovirus outbreak may theoretically occur at any probability, our findings, which are based on a comparison of model predictions with a limited number of observed outbreaks, suggest that there is little risk of an outbreak when the NORF model predicted probability is < 0.5, and a high likelihood of an outbreak when the predicted probability is > 0.6.

Although it has been widely reported that norovirus outbreaks exhibit strong seasonality, with the outbreak peak occurring commonly in winter (Wang and Deng 2012; Westrell et al. 2010), to our knowledge, this is the first time that gage height has been identified as an environmental factor associated with oyster norovirus outbreaks. The practical significance of this finding is that extremely low tide (or gage height) in winter (during low temperature) could be used as an indicator of norovirus outbreaks.

Our findings also suggest that norovirus outbreaks generally occur when extremely low gage height occurs in combination with low water temperature, low salinity, strong offshore wind, and heavy antecedent rainfall. Daily change in gage height was also a predictor, which may reflect an effect of the minimum daily gage height. The daily minimum water temperature was the second most influential predictor. Low temperature may cause the pore size of oyster gills to contract, thereby increasing the concentration of norovirus particles that are larger than the contracted pore size (Hopkins 1935). Because wind (the third environmental predictor) is defined as the product of wind speed and wind direction, both the speed and the direction affect norovirus outbreaks. As previously noted, low salinity may enhance the binding of viruses to fine sediment particles (Maalouf et al. 2010), which may help virus particles persist in an infectious state for several months. The fifth predictor included in the NORF model was rainfall on the ninth day before a norovirus outbreak. This suggests the possibility of a 9-day time lag between the release of virus from norovirus sources, such as inadequately treated/untreated sewage from failing wastewater treatment plants and faulty septic systems (Burkhardt and Calci 2000; Flannery et al. 2012; Goblick et al. 2011; Rajko-Nenow et al. 2013; Wang and Deng 2012), and the contact with an oyster-growing area. The specific time lag for a given location is likely to depend on the size of drainage basin and the locations of the stations used to measure rainfall for a given oyster growing area.

The potential significance of the NORF model is that it may allow oyster norovirus outbreaks to be predicted in advance (if forecasting data are available), or at the onset of outbreak, making it possible to prevent or at least reduce the risk of norovirus to human health, and costly oyster recalls. Potential users of the NORF model include, but are not limited to, state public health agencies and federal public health and food safety agencies. If the NORF model produces an alert, a responsible agency would need to take water and oyster samples from the high-risk area to confirm the alert, in which case the infected oyster-growing area could be closed.

## Conclusions

We developed a probability-based model for predicting oyster norovirus outbreaks. The NORF model is based on five independent predictors, including gage height, temperature, salinity, rainfall, and wind. The model was developed using the Artificial Neural Network (ANN) Toolbox in the MATLAB program and 14 years of historical data collected from Louisiana oyster-harvesting areas along the Gulf of Mexico. The NORF model was validated with 7 additional years of data that were not used in the model development. Our findings suggest that oyster norovirus outbreaks are predictable; in our study area, the NORF model predicted historical outbreaks when the estimated probability was > 0.6, whereas no outbreak occurred when the probability was < 0.5. However, more outbreak data are needed to confirm the threshold probability for norovirus outbreaks. Gage height and temperature were the most important environmental predictors of oyster norovirus outbreaks, whereas wind, rainfall, and salinity also predicted norovirus outbreaks. Although the NORF model was specifically developed for oyster-harvesting areas along the Louisiana Gulf coast, the methods and particularly the environmental variables presented in this paper may be generally applicable to oyster-harvesting waters in other regions. If the model is confirmed for Louisiana and other areas, it may provide an effective means to predict potential oyster norovirus outbreaks in advance or at the onset of outbreaks, making it possible to prevent or at least reduce the risk of norovirus to human health and costly oyster recalls.
